# Finite element analysis of maxillary arch distalization using skeletal anchorage at three different application regions

**DOI:** 10.1038/s41598-025-10035-9

**Published:** 2025-07-05

**Authors:** Fırat Oğuz, Samet Özden

**Affiliations:** Department of Orthodontics, Faculty of Dentistry, Inonu University, Malatya, Turkey

**Keywords:** FEM, Distalizing, Orthodontic mini-implant, Anchorage, Anatomy, Medical research, Engineering, Materials science

## Abstract

This study aimed to evaluate total maxillary arch distalization using three different skeletal anchorage systems—Mini Screw-Assisted Keleş Slider (MKS), infrazygomatic crest (IZC) screw, and maxillary tuberosity (MT) screw—through finite element analysis (FEA). A 3D cranio-maxillary model including dentition, periodontal ligament, and alveolar bone was constructed. For the MKS and IZC groups, forces were applied at three vertical heights (0 mm, 3 mm, and 6 mm apically), while the MT group involved three directional applications: buccal, palatal, and combined bucco-palatal. This design yielded nine distinct simulation scenarios. Tooth movements were assessed along the x (transverse), y (sagittal), and z (vertical) axes, and Von Mises stress distributions were analyzed in surrounding structures. In the MKS group, the first scenario showed the greatest molar crown displacement, while the third had the highest root-level movement. The ninth MT scenario yielded the most palatal crown displacement of incisors, while the sixth IZC scenario showed the greatest root movement. Apical force applications (MKS and IZC at 3–6 mm) allowed controlled displacement suited for Class II Division 2 malocclusions. In contrast, archwire-level and MT scenarios produced patterns favorable for Class II Division 1 cases. Anchorage type and force direction significantly affected distalization outcomes.

## Introduction

Class II malocclusions are among the most common orthodontic anomalies, ranking second in prevalence across various populations, ethnicities, and age groups^1,2^. Treatment planning considers patient age, malocclusion severity, soft tissue condition, expectations, and compliance. While functional therapies suit growing patients, non-growing individuals may require maxillary premolar extractions or molar distalization^3^. Distalization can be achieved using extraoral or intraoral appliances^4^though extraoral anchorage depends on compliance and presents esthetic concerns^5,6^. In contrast, intraoral methods are favored for their reduced reliance on cooperation, superior esthetics, adaptability, and continuous force application^7^. To prevent anchorage loss in intraoral distalization, skeletal anchorage has been introduced^8^.

Keleş et al.^9^ modified their design by adding a palatal screw, creating the Mini Screw-Assisted Keleş Slider (MKS), which withstands heavy forces. Another method involves infrazygomatic crest (IZC) screws, which eliminate root contact risk and allow minimally invasive placement^10^. Maxillary tuberosity (MT) screws provide another option, applying force at the alveolar crest for added stability. Despite thin cortical bone in the maxillary tuberosity, this region offers minimal risk of molar root damage and enhances tooth movement^11,12^.

Understanding the biomechanical effects of orthodontic forces is crucial due to the complexity of tooth movement and tissue response. Stress distribution and displacement occur in surrounding tissues, but their clinical determination is challenging, necessitating various force analysis methods. Finite element analysis (FEA) is the most widely used technique^13^enabling the evaluation of stress and displacement in complex geometries and predicting treatment outcomes in a simulated environment. This predictive capability enhances clinical precision and reduces errors^14^.

Although various studies in the literature have investigated different types of maxillary molar distalization using FEA^15–19^there is no study to date that simultaneously examines the effects from the palatal, buccal, and alveolar crest levels, as performed in this study. By incorporating these three distinct levels of force application, this study provides a more comprehensive understanding of the biomechanical responses associated with maxillary molar distalization. This approach not only enhances the accuracy of stress and displacement predictions across multiple anatomical regions but also offers valuable insights for optimizing clinical protocols. Building on this gap in the literature, the aim of this study is to analyze the biomechanical effects of maxillary molar distalization using FEA by evaluating force application at three distinct anatomical levels: palatal, buccal, and alveolar crest. By investigating these regions simultaneously, this study seeks to provide a detailed understanding of stress distribution and displacement patterns, ultimately contributing to the development of more precise and effective orthodontic treatment strategies.

## Materials and methods

### Ethical approval and study design

Ethical approval for this study was obtained from the Non-Interventional Clinical Research Ethics Committee of Inonu University (decision number 2023/4835, dated 2023).

**Fig. 1:**
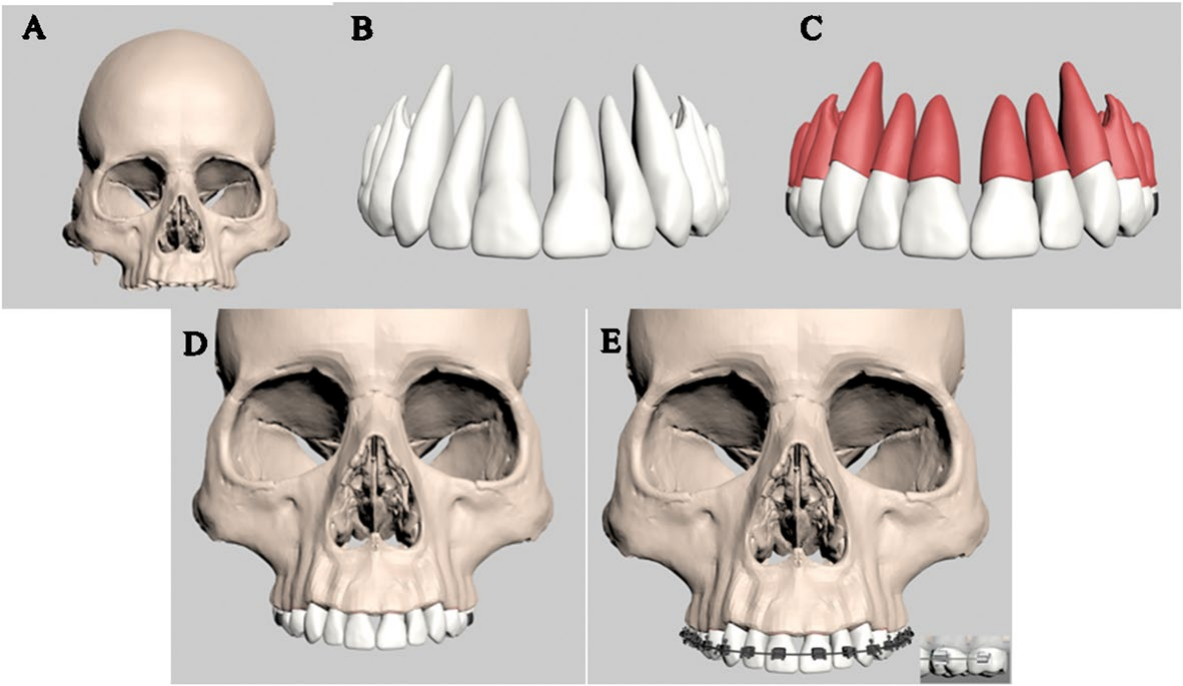
(A) Digital reconstruction of the craniomaxillary complex, (B) Maxillary dentition model, (C) Maxillary dentition model incorporating the PDL, (D) Study model of the dentition, (E) Study model of the dentition with brackets and molar tubes placement.

To construct the finite element model, the craniofacial complex, including dental structures and the periodontal ligament (PDL), was geometrically modeled using CBCT data from an edentulous maxilla and 3D-Doctor software (Able Software Corp., MA, USA). Teeth were modeled according to Wheeler’s dental anatomy atlas^20^with positions and angulations defined using the “Broad” arch form (Ormco, Glendora, CA, USA) and Andrews’ prescription values^21^. The PDL, a uniform 0.25 mm layer, was created at the tooth-cortical bone interface via slicing and designed to follow a path 1 mm apical to the cementoenamel junction (Fig. 1)^22^.

The finalized dental model incorporated an MBT bracket system with a 0.022” slot, including bands and molar tubes (Ormco Corp., Orange, CA, USA), along with a pre-fabricated 0.017” × 0.025” stainless steel archwire inserted into the braces^23^. To simulate total arch distalization, the archwire was cinched back distal to the second molar tube, with zero friction at the bracket-archwire interface to replicate free-sliding mechanics and ensure accurate force application (Fig. 1). Following the creation of the dental model with bonded brackets, soft tissue simulation was performed to replicate the gingival contours. The study evaluated three appliance designs, each subjected to forces from three vertical levels, resulting in nine unique scenarios for a comprehensive comparison of biomechanical effects across varying appliance configurations and force vectors.

### Computational framework and finite element modeling parameters

FEA in this study was conducted using Ansys (version 11; Ansys Inc., Canonsburg, PA, USA), a widely recognized software for complex biomechanical simulations. All analyses were performed on a same high-performance computer with (Processor Model, e.g., Intel Xeon^®^R CPU 3.30 GHz, 14 GB RAM, Windows 7 Ultimate Version Service Pack 1) ensuring accurate processing. Solid-tetrahedral elements were used to construct the finite element models, assuming homogeneity and isotropy. The number of elements and nodes is presented in Table 1, while material properties, including Elasticity Modulus and Poisson’s ratio, were defined based on literature values^17,24^ and detailed in Table 2. These parameters enabled FEA simulations to calculate tooth displacements and Von Mises stresses within the PDL, providing a detailed assessment of mechanical responses under varying conditions.

**Table 1:** Number of nodes and elements used in the scenarios.

	Nodes number	Elements number
1. Scenario	569,367	1,990,585
2. Scenario	577,323	2,023,634
3. Scenario	603,774	2,071,198
4. Scenario	494,520	1,729,114
5. Scenario	497,511	1,735,654
6. Scenario	497,488	1,735,531
7. Scenario	496,838	1,725,082
8. Scenario	496,838	1,725,082
9. Scenario	496,838	1,725,082

**Table 2:** Young’s modulus and poisson’s ratio of materials.

	Young’s modulus (MPa)	Poisson’s ratio
Cortical bone	13,700	0.3
Titanium	110,000	0.35
Acrylic	3000	0.35
Stainless Steel	200,000	0.3
Tooth	18,600	0.31
Periodontal ligament	0.68	0.45

### Computational modeling of distalization mechanics

#### Modeling of the Mini Screw-Assisted Keleş slider (MKS) appliance

**Fig. 2:**
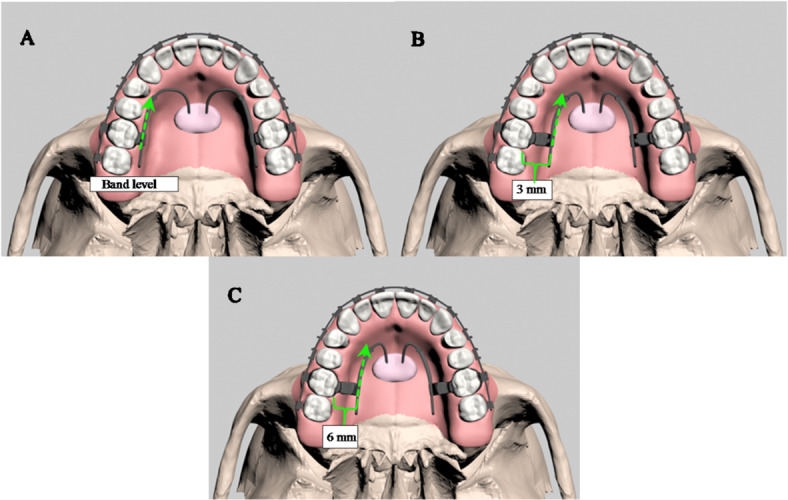
(A) Occlusal view of the MKS appliance applied at a 0 mm molar band level, (B) Occlusal view of the MKS appliance applied at a 3 mm vertical level, (C) Occlusal view of the MKS appliance applied at a 6 mm vertical level.

In the appliance design phase, the components of the MKS appliance ( the activator tube, stainless steel wire, nickel-titanium open-coil spring, gurin lock, and acrylic button) and two mini-screws measuring 1.5 mm in diameter and 8 mm in length (Bomei, Taiwan) were scanned with SmartOptics 3D scanner (Sensortechnik GmbH, Bochum, Germany) and subsequently modeled. In the MKS group, skeletal anchorage was established with two mini-screws placed bilaterally near the median palatal suture, 7–8 mm posterior to the incisive foramen and aligned with the third palatal rugae. Their heads were embedded in an acrylic structure for secure integration. Distalization forces of 400 g were applied to the first molars via the activator tube at three vertical levels (0 mm, 3 mm, and 6 mm), generating three finite element models to evaluate the biomechanical effects of force application across varying heights (Fig. 2).

#### Modeling of the Infra-Zygomatic crest (IZC) screw application

**Fig. 3:**
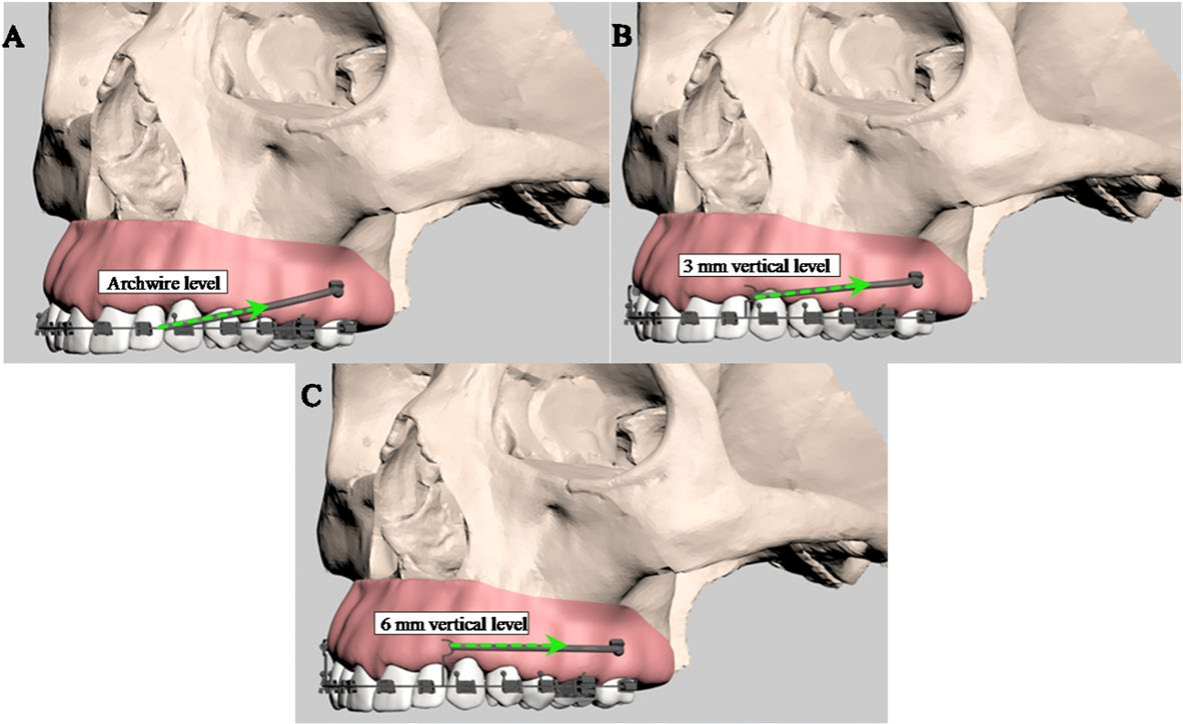
(A) Side view of the model with a closed coil spring applied at a 0 mm archwire level from the IZC screw, (B) Side view of the model with a closed coil spring applied at a 3 mm vertical level from the IZC screw, (C) Side view of the model with a closed coil spring applied at a 6 mm vertical level from the IZC screw.

In the IZC group, skeletal anchorage was established using a stainless steel infrazygomatic crest (IZC) screw (2 mm × 12 mm; OrthoBoneScrew, Newton’s A Ltd, Taiwan) integrated into the finite element model (Fig. 3). Prior to integration, the screw, closed-coil springs and rigid stainless steel retraction hooks were scanned and digitally modeled. Following Liou et al.‘s methodology^25^the screw was inserted at the mesiodistal midpoint of the maxillary first molar, angled 55°–70° to the occlusal plane, and positioned 14–16 mm apical to it. Distalization forces of 400 g were applied via closed-coil springs attached to rigid 0.036” stainless steel retraction hooks at three vertical levels (0 mm, 3 mm, and 6 mm) (Fig. 3).

#### Modeling of the maxillary tuber (MT) screw application

**Fig. 4:**
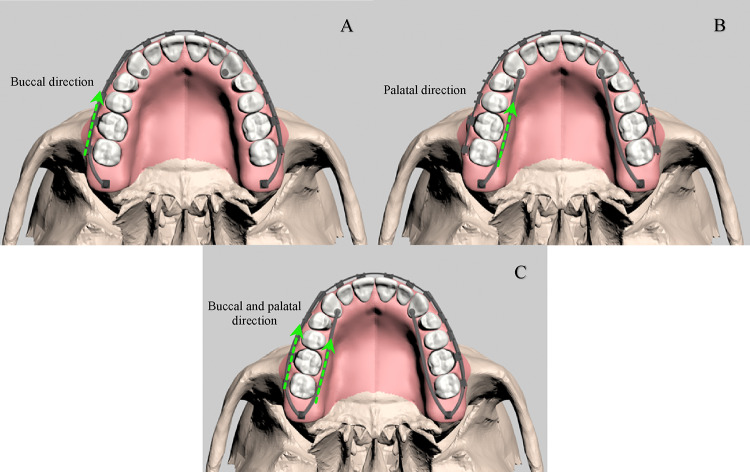
(A) Occlusal view of the model with buccal force applied to the maxillary canine from the MT screw, (B) Occlusal view of the model with palatal force applied to the maxillary canine from the MT screw, (C) Occlusal view of the model with both buccal and palatal forces applied to the maxillary canine from the MT screw.

In the MT group, skeletal anchorage was established using a 2 mm × 12 mm mini-screw (OrthoBoneScrew, Newton’s A Ltd, Taiwan), selected based on parameters from Paredes et al.^26^. The screw dimensions were determined considering maxillary tuberosity bone density and its anatomical relationships. Prior to integration, the screw, closed-coil springs and button were scanned and digitally modeled. Positioned at a 20°–40° angle to the occlusal plane, the mini-screws generated distalization forces via closed-coil springs delivering 400 g, connecting the screw to the maxillary canine. Force application was analyzed at three levels: buccal, palatal, and a combination of both (Fig. 4).

#### Experimental scenario design

Based on the configurations described under the previous headings, the scenarios defined in this study are as follows:

**Group 1 (MKS Appliance Group)**:

Scenario 1: 0 mm apically from the first molar band level.

Scenario 2: 3 mm apically from the first molar band level.

Scenario 3: 6 mm apically from the first molar band level.

**Group 2 (IZC Screw Group)**:

Scenario 4: 0 mm apically from the archwire level.

Scenario 5: 3 mm apically from the archwire level.

Scenario 6: 6 mm apically from the archwire level.

**Group 3 (MT Screw Group)**:

Scenario 7: Buccal button level of the maxillary canine.

Scenario 8: Palatal button level of the maxillary canine.

Scenario 9: Both buccal and palatal button levels of the maxillary canine.

**Fig. 5:**
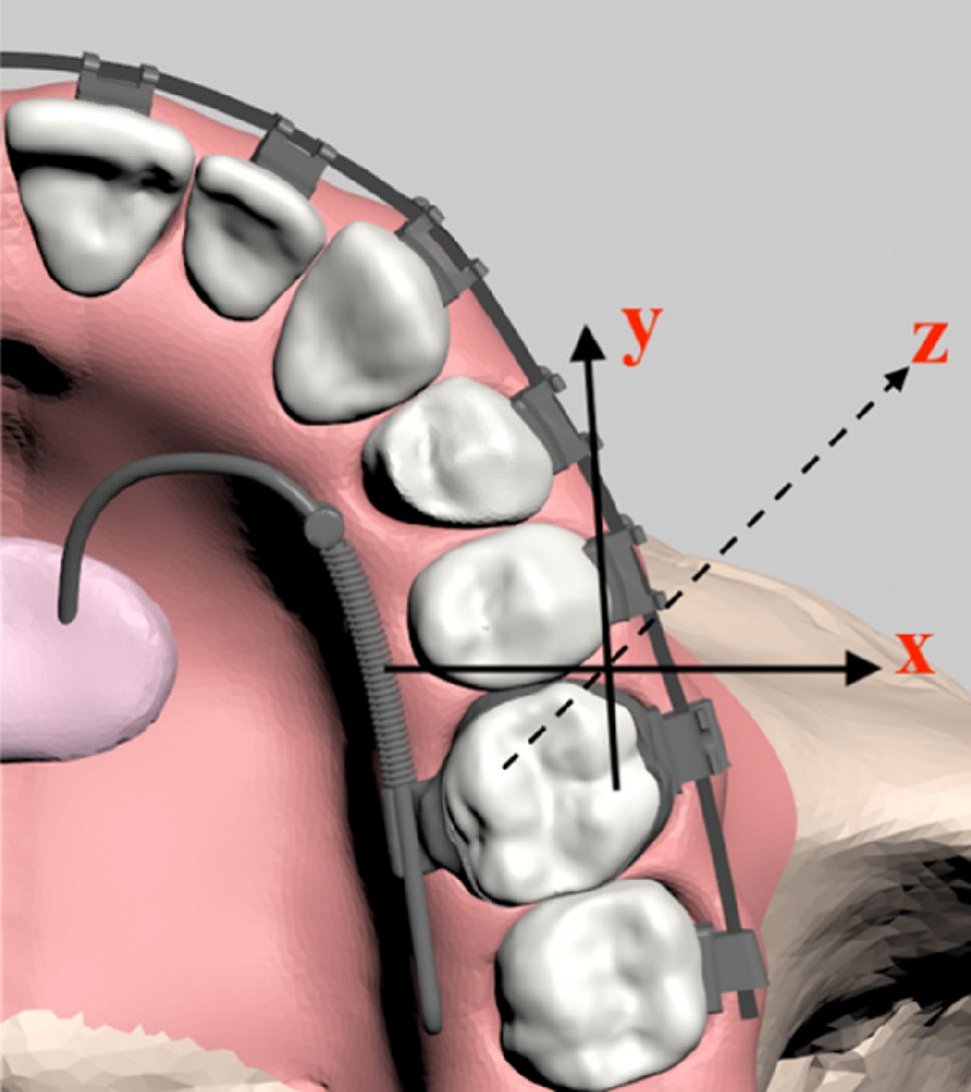
Coordinate system. x-axis: (+) buccal, (−) palatal direction; y-axis: (+) posterior, (−)anterior direction; z-axis: (+) superior, (−) inferior direction.

In all generated scenarios, tooth displacements were meticulously analyzed across three distinct axes: X, Y, and Z, ensuring a comprehensive assessment of movement patterns (Fig. 5).

## Results

**Fig. 6:**
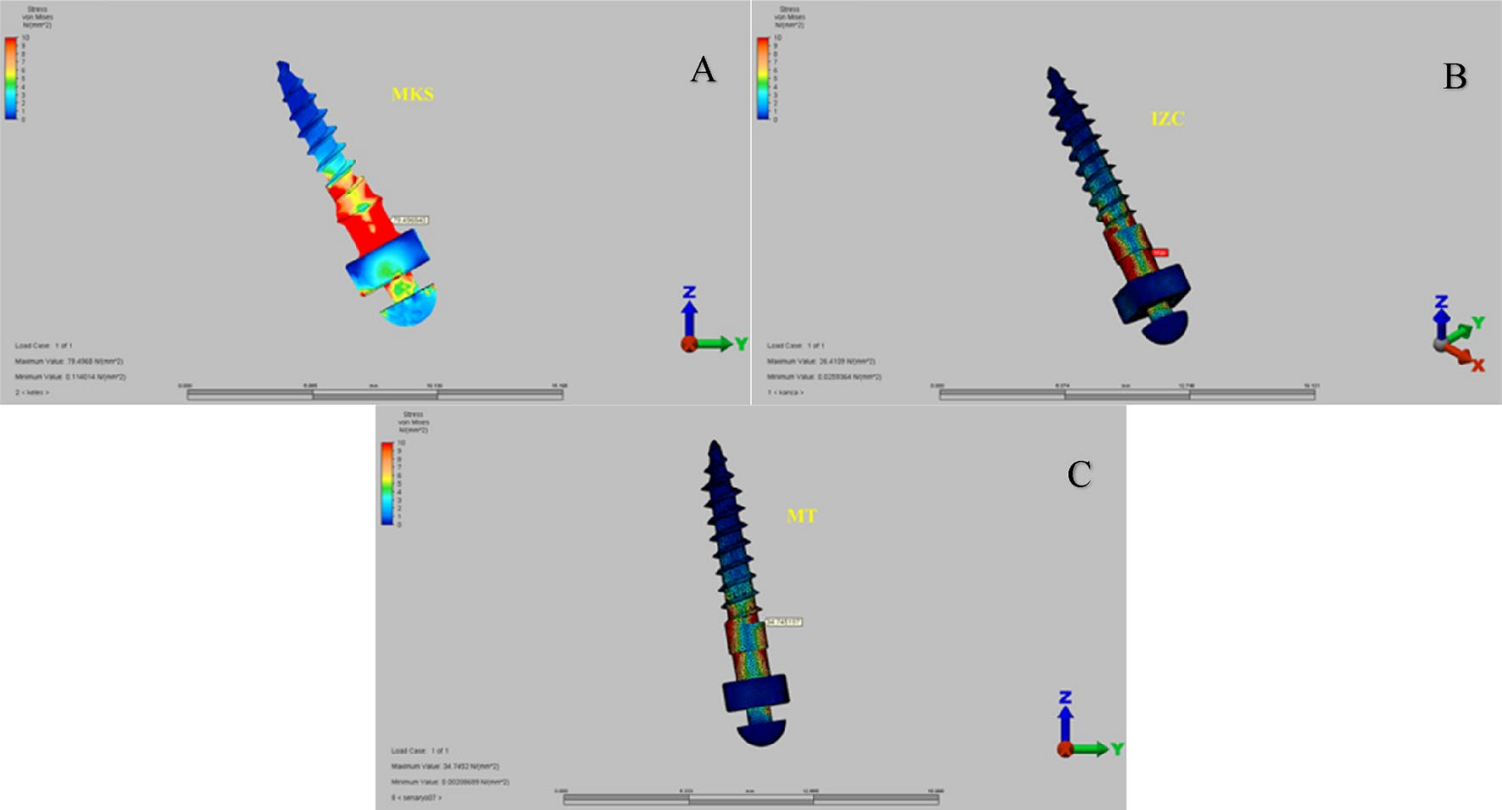
Von Mises stress distribution around (A) MKS appliance screw, (B) IZC screw, (C) MT screw.

Von Mises stress distributions around the mini-implants revealed that the highest stress concentrations were localized at the “neck” region of the mini-implants across all three groups (Fig. 6).

**Fig. 7:**
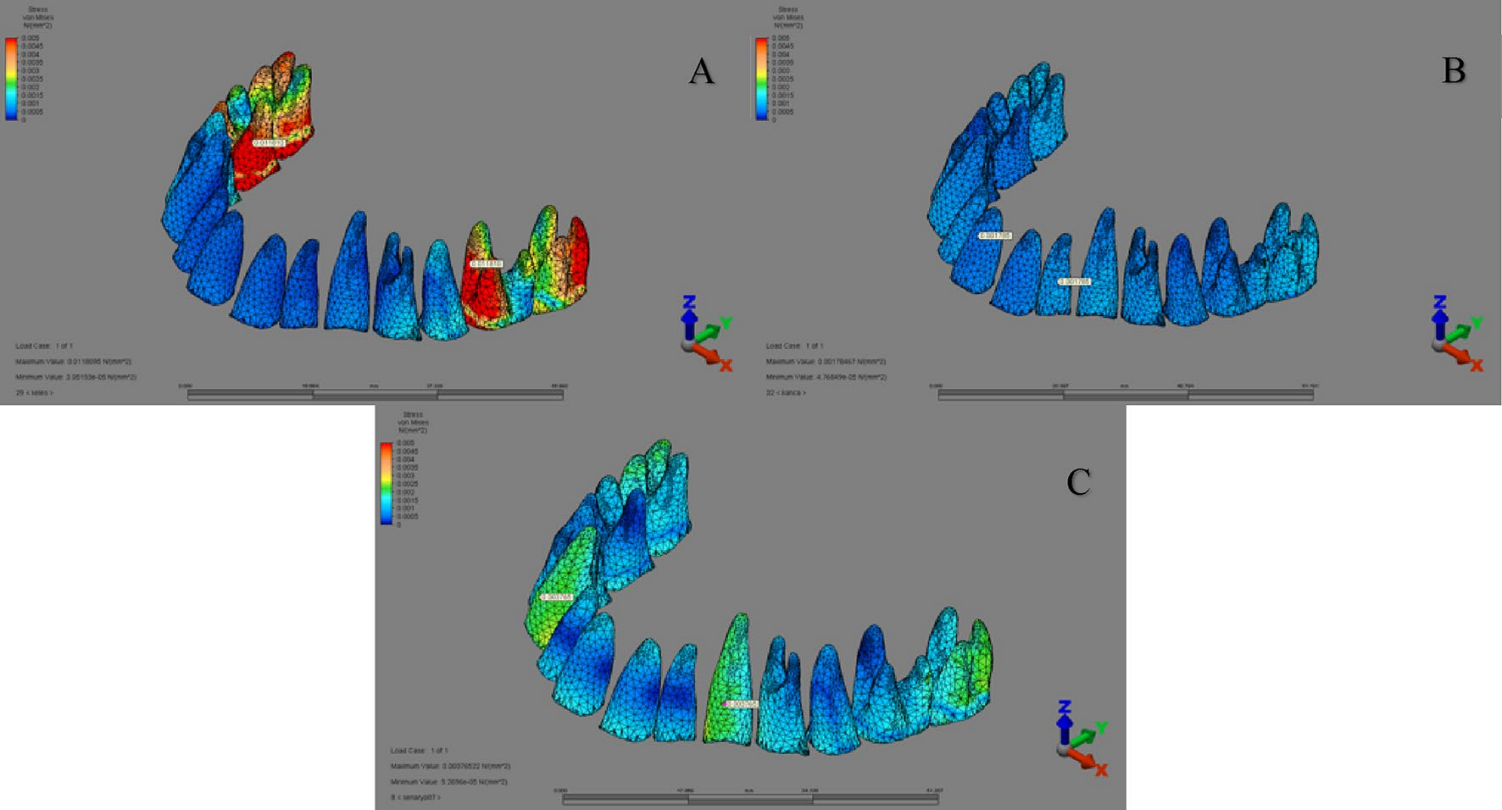
Von Mises stress distribution around the roots (A) MKS appliance, (B) IZC screw, (C) MT screw.

Analysis of Von Mises stress distributions within the tooth roots revealed distinct patterns across groups. In the ‘MKS group’ (1st–3rd scenarios), the highest stress concentration was localized at the palatal root of the first molar. In the ‘IZC group’ (4th–6th scenarios), maximum stress was observed at the lateral incisor root, while in the ‘MT group’ (7th–9th scenarios), the greatest stress levels were identified in the canine and adjacent teeth (Fig. 7).

**Fig. 8:**
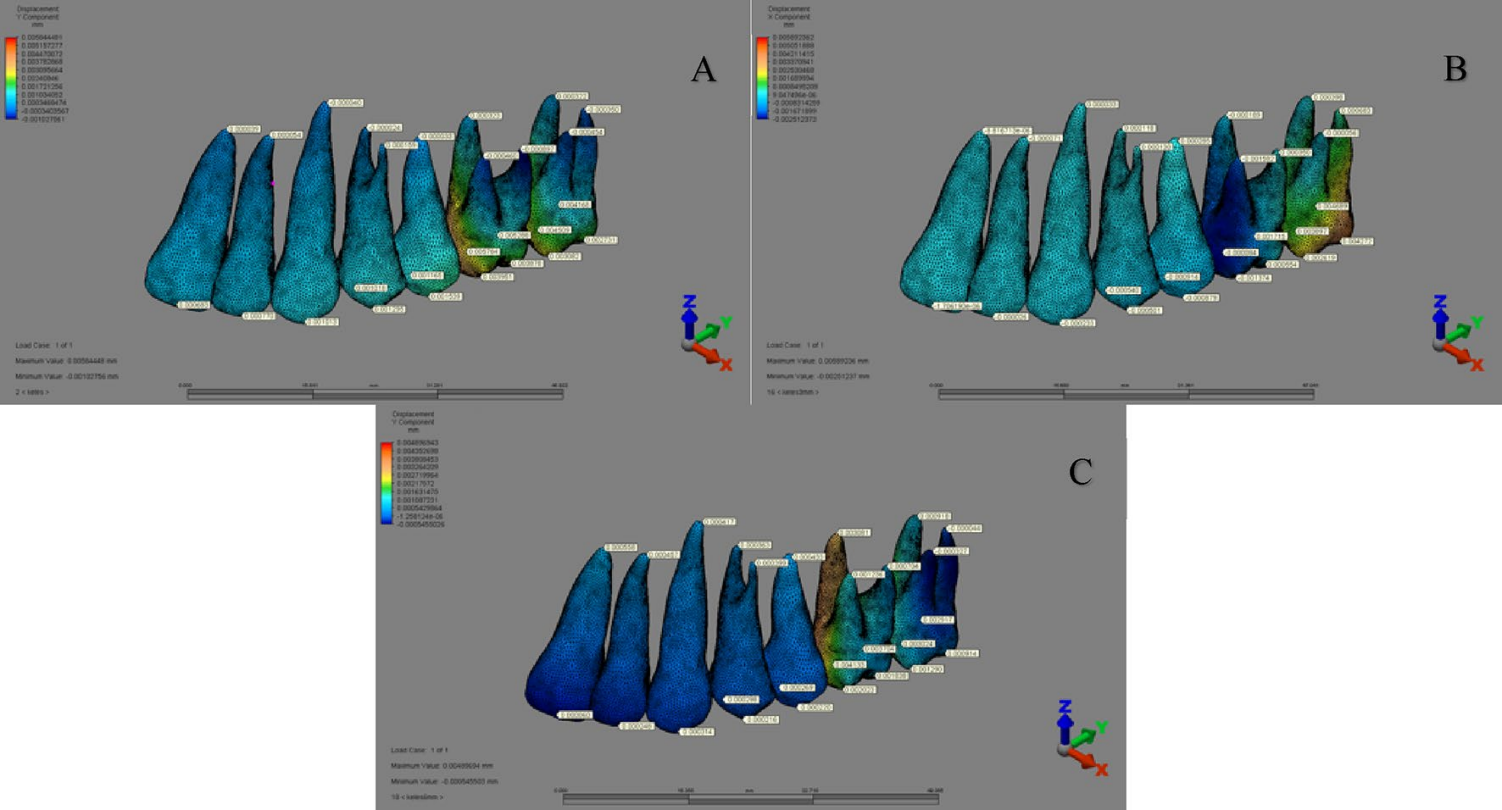
Displacement distribution along the Y-axis in the MKS group for (A) 1st scenario, (B) 2nd scenario, and (C) 3rd scenario.

When the displacements in the teeth were analyzed, it was observed that:

In the ‘MKS group’ (1st–3rd scenarios), the highest displacement occurred at the distopalatal cusp tip of the second molar in the buccal direction (X-axis), the mesiopalatal cusp tip of the first molar in the distal direction (Y-axis), and the distobuccal cusp tip of the second molar in the apical direction (Z-axis) (Fig. 8).

**Fig. 9:**
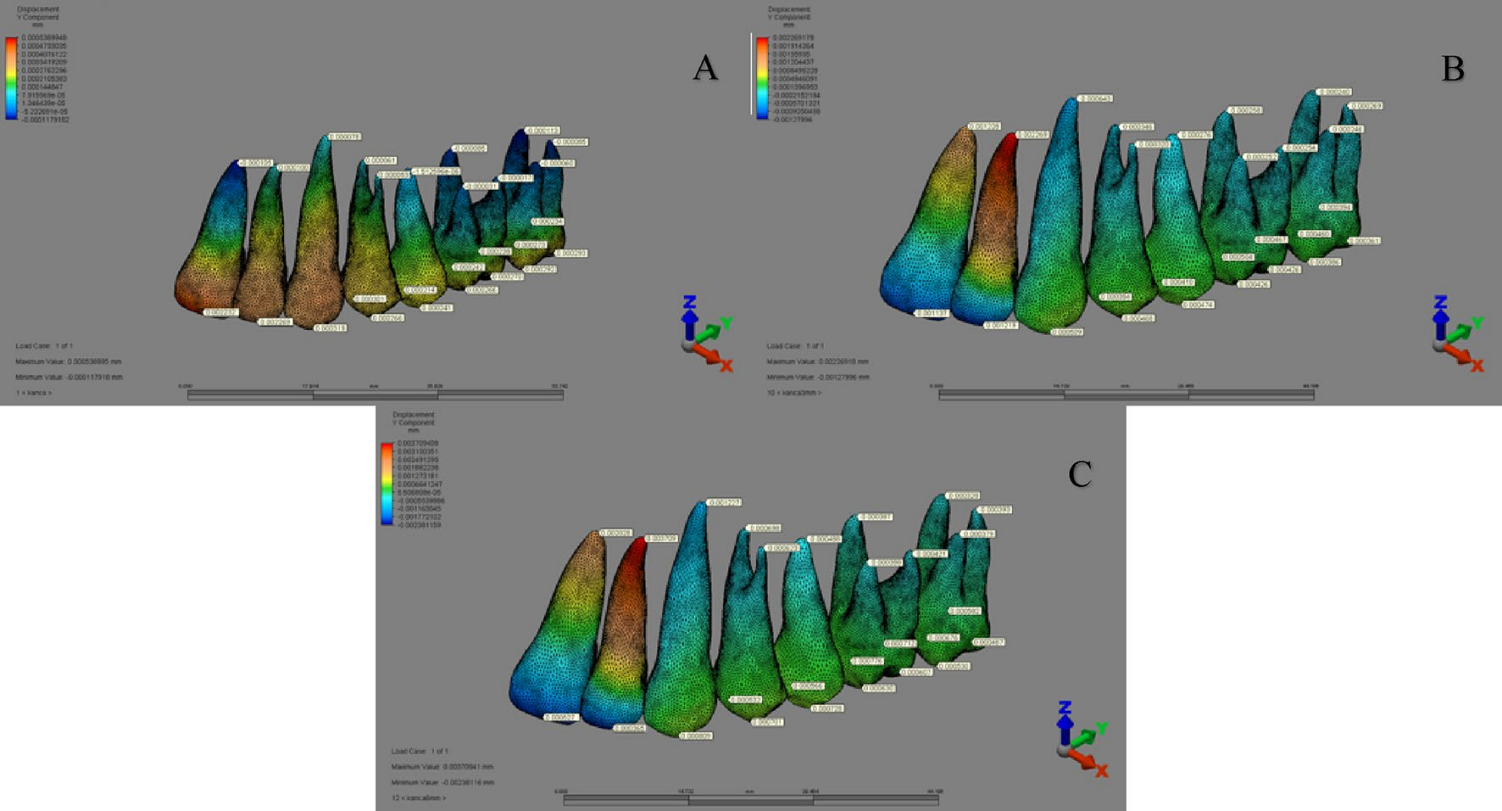
Displacement distribution along the Y-axis in the IZC group for (A) 4th scenario, (B) 5th scenario, and (C) 6th scenario.

In the ‘IZC group’, displacement varied across scenarios: in the 4th scenario, maximum displacement occurred at the palatal apex of the first premolar (X-axis), the incisal edge of the lateral incisor (Y-axis), and the distopalatal cusp tip of the second molar (Z-axis). In the 5th and 6th scenarios, the highest displacement was noted at the canine apex in the buccal direction (X-axis), the lateral incisor apex in the palatal direction (Y-axis), and the incisal edge of the central incisor in the apical direction (Z-axis) (Fig. 9).

**Fig. 10:**
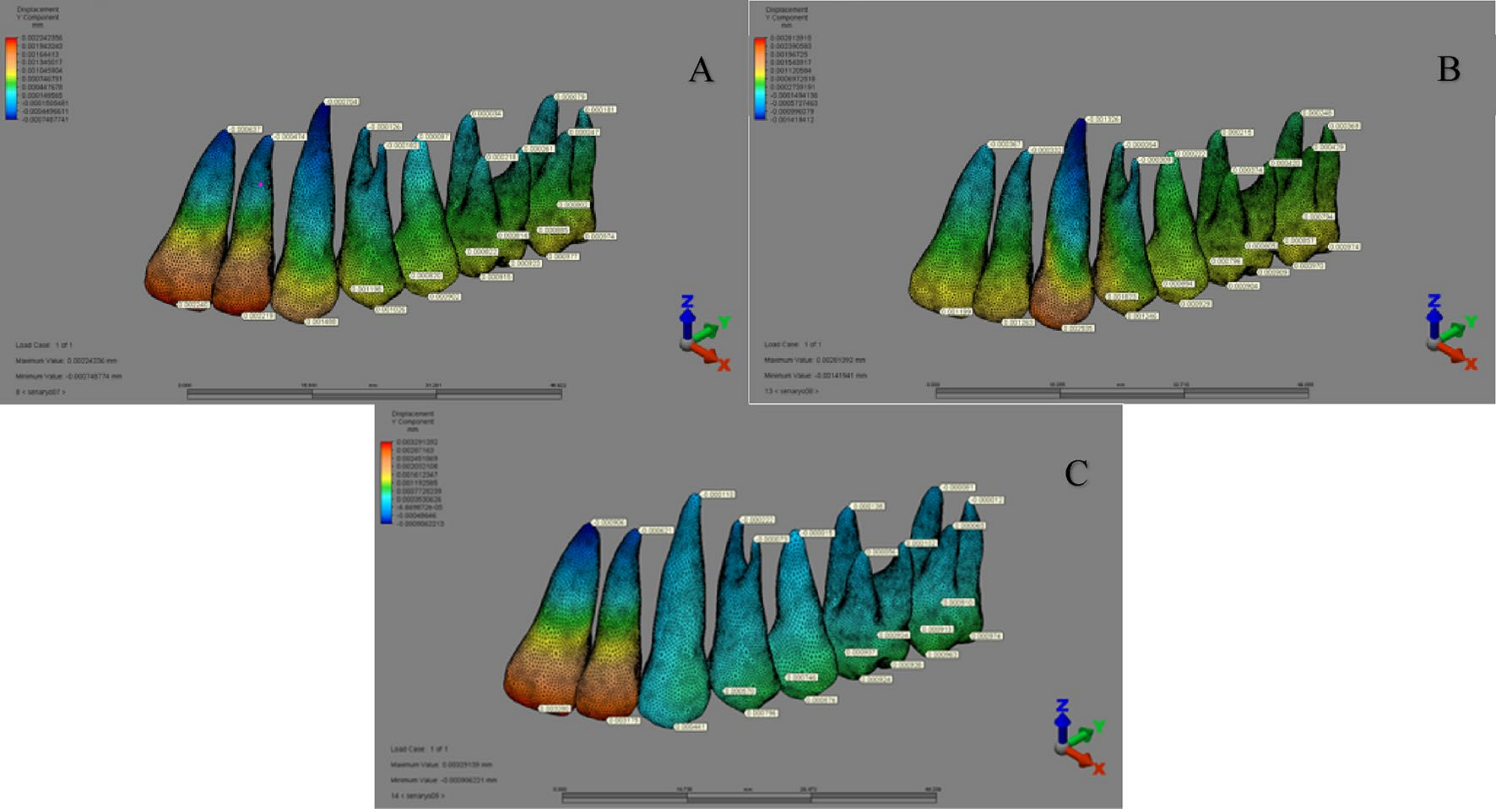
Displacement distribution along the Y-axis in the MT group for (A) 7th scenario, (B) 8th scenario, and (C) 9th scenario.

In the ‘MT group’, displacement assessments showed that in the 7th and 8th scenarios, the greatest displacement occurred at the buccal cusp tip of the second premolar (X-axis), while in the 9th scenario, it was at the canine cusp tip in the buccal direction. Along the Y-axis, the highest displacement was seen at the incisal edge of the central incisor in the palatal direction (7th and 9th scenarios) and at the canine cusp tip in the distal direction (8th scenario). On the Z-axis, the greatest displacement occurred at the canine cusp tip (7th and 8th scenarios) and at the incisal edge of the central incisor in the coronal direction (9th scenario) (Fig. 10).

## Discussion

The effectiveness of distalization depends on precise force application and strategic anchorage positioning. In continuous archwire systems, inadequate anchorage can cause unpredictable tooth movements due to complex force distribution^15,27^. While individual tooth movement is predictable, fixed arch systems pose challenges due to their statically indeterminate nature, leading to non-linear displacement patterns. Although mathematical models provide insights, clinical decisions rely on comprehensive dentition assessments to optimize distalization and treatment planning.

Initially developed for engineering, FEA has been adapted to dentistry with technological advancements, enabling precise analysis of complex biomechanical interactions in orthodontics^28–30^. This transition highlights the role of computational methods in understanding orthodontic forces. To perform FEA, study models are first created, incorporating scanned dental structures such as brackets, molar tubes, archwires, mini-screws, acrylic Nance button, palatal appliance components, and closed-coil springs to accurately simulate clinical conditions. All structures, including the PDL, were modeled as homogeneous, isotropic, and linearly elastic to simplify computations while ensuring reliability.

Additionally, in our study, solid-tetrahedral elements were used to construct the mathematical model, as reported by Gautam et al.^31^ to be ideal for simulating the complex structures of teeth and surrounding tissues. This method enables precise stress distribution and tooth movement analysis, providing valuable insights into biomechanical responses under orthodontic forces. The ability of FEA to generate detailed, reproducible data makes it indispensable for understanding dental mechanics and optimizing treatment strategies.

The position of force application is crucial in tooth distalization, as buccal and palatal forces create distinct movement patterns that affect precision, treatment duration, and effectiveness. Distalizing the entire maxillary dentition increases biomechanical complexity due to tooth and supporting structure interactions. The MKS, IZC, and MT screws apply force from different vectors—MKS palatally, IZC buccally, and MT from both—each producing unique effects. This study evaluates maxillary arch distalization by analyzing tooth movements and PDL stresses under buccal, palatal, and combined force applications while also assessing root resorptive effects to mitigate complications. By comprehensively examining distalization biomechanics, this research offers valuable insights for optimizing treatment strategies and improving clinical outcomes.

As expected, our findings confirmed that variations in bucco-lingual and vertical force application resulted in distinct tooth movement patterns and stress distributions, highlighting the complexity of distalization. To achieve total maxillary arch distalization, three approaches were utilized, each with unique biomechanical and clinical benefits. The MKS appliance provides stable anchorage through dense palatal bone, while its acrylic base protects mini-screws from external influences^9^. The IZC screw, anchored in the thick cortical bone of the infrazygomatic crest, ensures strong stability with minimal risk of screw failure, reducing root damage and enhancing clinical efficiency^32^. The MT screw facilitates broader distalization with minimal root interference and supports en masse retraction, demonstrating favorable clinical outcomes that reinforce its effectiveness in maxillary distalization^33,34^.

A comprehensive literature review was conducted to determine the optimal force magnitude for total maxillary distalization, revealing a range of recommendations. Chang et al.^10^ suggested forces between 8 and 14 oz (227–400 g), while Janssens et al.^35^ applied 150–200 centinewtons per mini-screw palatally and buccally, generating a unilateral force of 300–400 centinewtons (305–407 g). Bechtold et al.^36^emphasized that 400 g is particularly effective for efficient distalization. Based on these findings, our study standardized the force magnitude at 400 g unilaterally across all scenarios to ensure consistency and clinical relevance.

The analysis of Von Mises stress distributions around the mini-screws demonstrated that the highest stress concentrations were consistently localized at the “neck” region across all three groups. This observation aligns with the findings of Ammar et al.^37^who utilized FEA to evaluate distalization with mini-screw anchorage and reported that the maximum stress was concentrated in the neck region of the screws. The MKS group exhibited the highest stress at the mini-screw neck, likely due to its smaller screw dimensions (1.5 mm × 8 mm) compared to the larger screws (2 mm × 12 mm) in other groups, as studies confirm that increasing implant diameter reduces stress^38^. Additionally, the acrylic design may have concentrated forces on the mini-screws, further contributing to higher stress levels. These findings emphasize the combined impact of screw size, appliance design, and biomechanical factors on stress distribution, highlighting the need for optimal screw selection and appliance configuration to ensure stable anchorage.

When comparing sagittal distalization among the three groups, notable differences in molar displacement patterns emerged at both the crown and root levels, despite identical force magnitudes. The first MKS scenario exhibited the greatest crown-level distalization, while the third MKS scenario showed the highest root displacement, likely due to variations in force vectors. In the MKS group, force was applied palatally at the first molar, whereas in the IZC and MT groups, it originated buccally from the anterior region. The greater distalization observed in the MKS group may result from palatal anchorage advantages, as studies suggest that palatal-supported appliances apply forces closer to the center of resistance, promoting bodily movement with reduced tipping^39^. Wilmes et al.^40^ conducted a clinical study on the Beneslider appliance and reported that the molars were distalized with only 1.9 degrees of tipping. They attributed this effect to the force vector passing close to the center of resistance of the molar.

Yu et al.^41^found that apical force enhances root movement and minimizes tipping, whereas coronal force increases crown displacement and tipping. Similarly, Antonarakis et al.^39^ reported that buccal distalization appliances apply forces farther from the center of resistance, leading to greater tipping, while palatal mechanics facilitate more parallel distalization, a finding further supported by Kook et al.^42^who optimized palatal anchorage plates to refine force direction and minimize tipping. Kook et al.^42^reported in their clinical study that by positioning the appliance closer to the palatal vault, the force vector was brought closer to the center of resistance, resulting in molar distalization with minimal tipping. On the other hand, in the clinical study conducted by Aslan et al.^43^it was reported that a similar amount of molar distalization was achieved using both the palatal anchorage appliance (MSP) and the IZC method. In the MSP group, an average distalization of 3.52 ± 0.76 mm was achieved in 8.71 ± 2.02 months, whereas in the IZC group, a comparable amount (3.50 ± 0.74 mm) required a longer duration of 9.7 ± 2.5 months. Moreover, although significant molar tipping was observed in both groups, the mean tipping angle was 10.21° in the MSP group and 11.41° in the IZC group, indicating that the palatal anchorage system resulted in relatively less tipping. These findings highlight that force application location is a critical determinant of distalization patterns, with root movement in the MKS group supporting the notion that forces applied closer to the center of resistance enable more controlled and efficient distalization than buccal applications.

When comparing incisor movement across the three groups, distinct displacement patterns emerged, with the greatest palatal crown displacement in the ninth MT scenario and the highest root displacement in the sixth IZC scenario, highlighting the impact of force application location and direction on anterior tooth movement during total arch distalization. Previous studies indicate that force application height influences incisor displacement; one study found that crowns moved most when force was applied at the archwire level^16^while Rosa et al.^44^ reported that IZC screw forces transmitted through an archwire hook caused lingual crown tipping and buccal root movement. Similarly, Sung et al.^27^ observed that lower force application resulted in palatal crown movement, whereas higher vectors induced lingual root and labial crown displacement. Our findings align with these conclusions, emphasizing the role of vertical force application in anterior movement patterns. The significant crown movement in the MT group, greater than in the fourth IZC scenario, may be attributed to its larger horizontal force vector and alveolar crest positioning, which facilitated more parallel force transmission. These results underscore the importance of strategic force application and anchorage selection to optimize incisor movement while minimizing undesirable tipping.

When evaluating vertical tooth movements, assessing overall arch movement is more clinically meaningful than focusing solely on individual extrusion or intrusion. In cases where a rigid archwire stabilizes the arch, analyzing occlusal plane rotation—clockwise or counterclockwise—provides key insights into the maxillomandibular effects of treatment and clarifies biomechanical responses during maxillary distalization. Comparing the three groups, a counterclockwise occlusal plane rotation was observed in the MKS and IZC groups as the force vector moved apically, while a clockwise rotation occurred in the MT group, where force was applied at the crown level. Wu et al.^19^ reported that applying force to high-level retraction hooks flattens the occlusal plane by directing the force above the center of resistance, increasing incisor intrusion, a finding consistent with Khan et al.^16^. In the IZC group, particularly in the sixth scenario, incisor intrusion was more pronounced, whereas the MT group exhibited predominant extrusion, likely due to force application at the archwire level and below the center of resistance. Wu et al.^19^ previously noted that lower retraction hook placement increases maxillary incisor extrusion, supporting our findings. In the posterior region, the MKS group showed the greatest vertical displacement, with second molar intrusion and first molar extrusion, likely due to the force vector acting below the arch’s center of rotation. This clockwise rotation led to anterior and first molar extrusion, while the second molar intruded—a pattern consistent with previous studies^15,17,41^. Our findings on vertical movement patterns highlight the critical role of force vector positioning in occlusal plane rotation, directly influencing treatment mechanics. Thus, careful control of force direction and magnitude is essential in maxillary distalization to optimize outcomes while minimizing unwanted vertical changes.

When evaluating transverse changes, distinct differences in arch expansion and contraction patterns emerged. In the molar region, the greatest expansion occurred in the MKS group, with the most pronounced widening in the third scenario, where force was applied 6 mm apically between the second molars, aligning with studies^5,17,41^ reporting that first molar distalization often induces second molar expansion due to force distribution. In the canine and premolar regions, the fourth IZC scenario exhibited the greatest expansion, likely due to direct force application from the IZC screw to the archwire^42,44^which may have induced a buccal flaring effect, particularly in anterior segments. Conversely, the sixth IZC scenario showed the most significant constriction, suggesting that vertical force position influences transverse movement, as retraction hook elongation may have altered force distribution, creating a distal deflection point and facilitating palatal movement. These findings underscore the intricate interplay between force height, archwire mechanics, and transverse displacement, emphasizing the need for precise force vector control in maxillary distalization.

A primary limitation of this study is the inability to fully replicate the complexity of a living organism in a digital environment. Although teeth, cortical and cancellous bone, periodontal ligament, mini-screws, and appliances were successfully modeled, the absence of muscle and soft tissue remains a constraint. Furthermore, the numerous biological variables influencing orthodontic tooth movement cannot yet be fully integrated into computational simulations due to technological limitations. Another drawback of finite element analysis is that displacement data reflect only the immediate response to applied forces, as FEA does not account for progressive biological adaptations over time. Therefore, non-linear analyses would be necessary to comprehensively evaluate the long-term effects of distalization.

## Conclusion

- Although the first scenario exhibited the greatest crown distalization, it primarily involved crown tipping, potentially increasing the risk of anchorage loss over time. In contrast, the third scenario demonstrated more controlled displacement with greater root movement, indicating improved long-term stability.
- In the third and sixth scenarios of the MKS and IZC groups, force application at 3 mm and 6 mm apically positioned the force vector closer to the center of resistance, reducing palatal movement of incisor crowns relative to their roots. This pattern may be advantageous in Class II Division 2 malocclusion, where maxillary arch distalization requires controlled incisor root movement.
- In the first and fourth scenarios of the MKS and IZC groups, as well as all MT group scenarios, incisor crowns exhibited greater palatal tipping than the roots, reducing incisor inclination. This movement may be beneficial in Class II Division 1 malocclusion, where maxillary arch distalization combined with anterior crown retraction helps manage excessive incisor inclination.
- Despite differences in distalization patterns across the three mechanics evaluated, all demonstrated effective maxillary arch distalization. Based on these findings, selecting appropriate anchorage systems and force vectors according to the patient’s sagittal, vertical, and transverse discrepancies is clinically important, as it can help clinicians optimize treatment strategies and achieve more predictable and stable outcomes.

## Data Availability

The original contributions presented in the study are included in the article, further inquiries can be directed to the corresponding author.
